# Impulsivity, suicide risk and cannabis consumption

**DOI:** 10.1192/j.eurpsy.2024.725

**Published:** 2024-08-27

**Authors:** E. Smaoui, D. Mnif, R. Ouali, R. Sellami, I. Feki, I. Baati, J. Masmoudi

**Affiliations:** ^1^Psychiatry, CHU hédi chaker, Sfax, Tunisia

## Abstract

**Introduction:**

Cannabis is the most widely consumed illegal drug in the world and one of the easiest to access. This drug provides a feeling of well-being and euphoria. However, frequent consumption is associated with several complications including increased impulsivity and an increased risk of suicidal behaviour.

**Objectives:**

Our objective was to study the link between cannabis consumption, impulsivity and suicide intentionality.

**Methods:**

We conducted a cross-sectional study, during the period from September 2020 to October 2021, among cannabis users consulting the Sfax Detoxification Center in Tunisia. Impulsivity was studied using the Barrat Impulsivity Scale (BIS 15) and suicide intentionality was assessed using the Suicide intent scale Beck; Pierce (SIS) in subjects with history of a suicide attempt.

**Results:**

We included 38 consumers. The average age is 26 years old and the sex ratio was 8.5 with an over-representation of men. The average BIS15 score was 38.2 ranging between 19 and 45. We have demonstrated that the higher the level of cannabis dependence, the higher the level of impulsivity. A high level of impulsivity was found in younger subjects (p=0,04) and with a low socio-economic level and unemployment (p =0,021). Suicidal intentionality, assessed in 10 patients with a history of suicide attempt, was low and intermediate in 40% and 60% of users respectively, which means a low to intermediate risk of subsequent completed suicide.

**Conclusions:**

Impulsivity is associated with aggressive behaviour, various accidents including motor vehicle accidents, more self-mutilation and a much greater risk of dying by suicide than the general population. Frequent cannabis use is also associated with increased risk of developing all types of suicidal behaviours independently of the existence of depressive symptomatology. Overall, it is important to take into account the issues of impulsivity and substance abuse in daily clinical work as they influence the level of dangerousness.

**Disclosure of Interest:**

None Declared

